# Supplementary figures and images for: On taming the effect of transcript level intra-condition count variation during differential expression analysis: A story of dogs, foxes and wolves
Source: PLoS One. 2022 Sep 22;17(9):e0274591. doi: 10.1371/journal.pone.0274591 (PMC9498955; doi:10.1371/journal.pone.0274591)

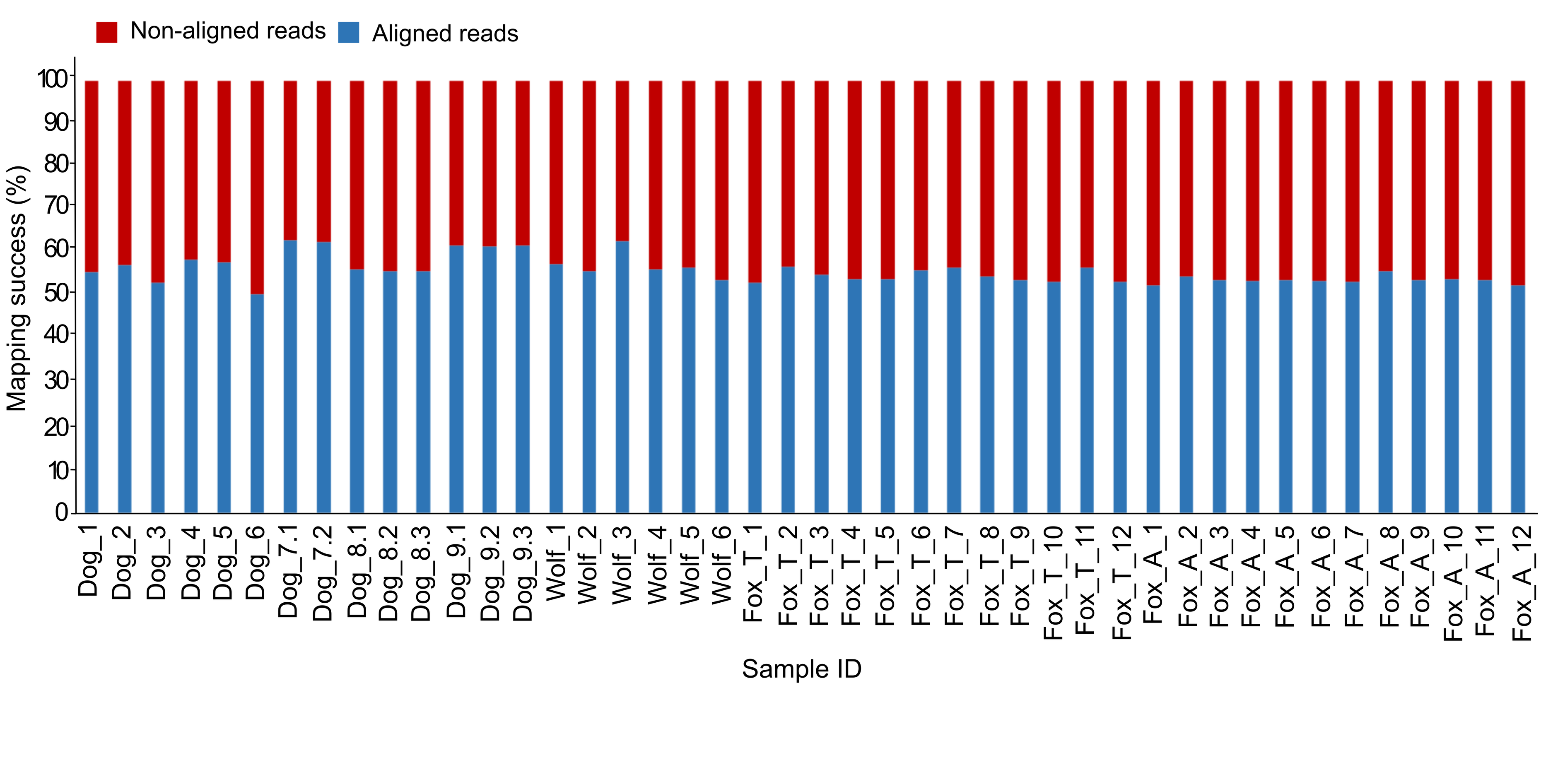

Supplement: S1 Fig — Mapping success rates (%) resulting from the alignment of the 44 samples used in this study to the complete dog transcriptome. For each sample, the percentage of aligned reads is presented by the blue bars, while the percentage of reads failing to map is represented in red (the number of raw reads is available in S1 Table). (TIF) [file pone.0274591.s001.tif]

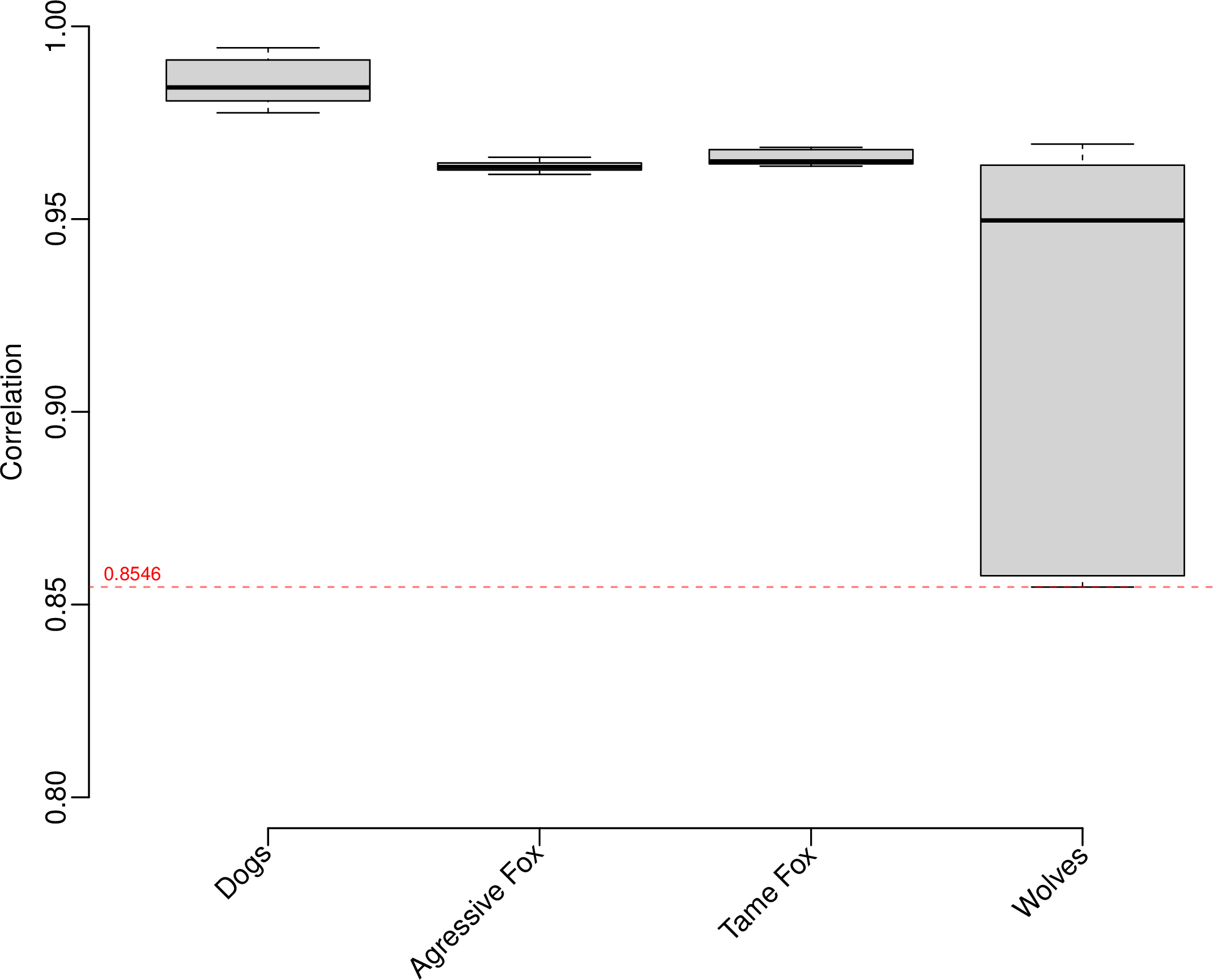

Supplement: S2 Fig — R2 values describing the linear correlation between each count dataset produced from the mapped datasets presented in S1 Fig and corresponding count extimates produced when pseudo-mapping the same RNA-Seq data to the complete dog transcriptome using kallisto. (TIF) [file pone.0274591.s002.tif]

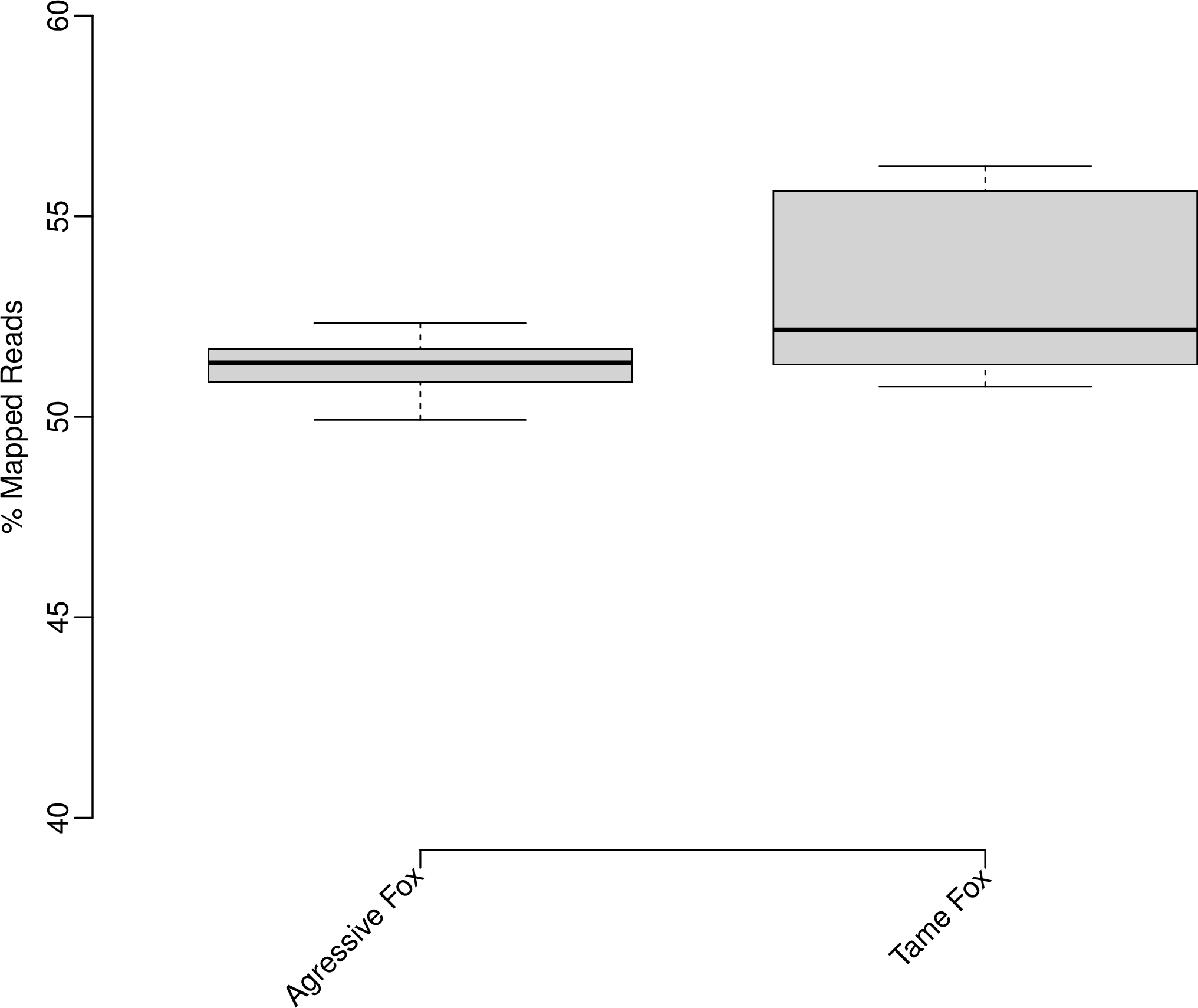

Supplement: S3 Fig — Read mapping rates achieved when mapping the fox RNA-Seq datasets to the fox reference transcriptome. (TIF) [file pone.0274591.s003.tif]

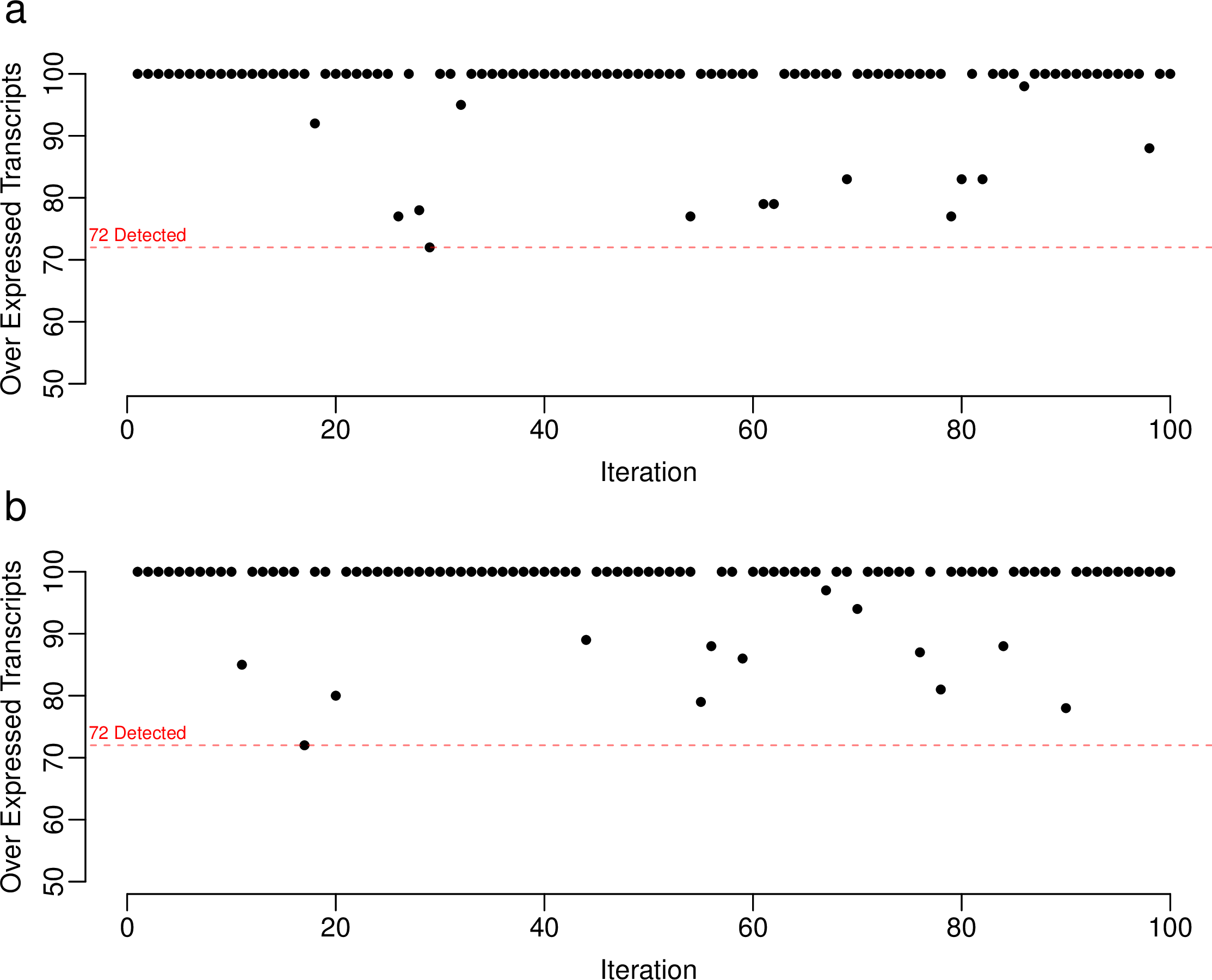

Supplement: S4 Fig — Across one hundred iterations the dots represent the number of transcripts identified as being over expressed between condition A and B. Each condition contained five replicates. (A) The one hundred transcripts selected for read over representation within replicates of condition B were maintained as constant and (B) the one hundred transcripts selected for read over representation within replicates of condition B were re-selected during each iteration. During each iteration the ten count datasets that were simulated each reflected even transcript coverage of 3 million read pairs with the exception of the one hundred transcripts selected for over representation in condition B whose count values were increase by a factor of two. (TIF) [file pone.0274591.s004.tif]

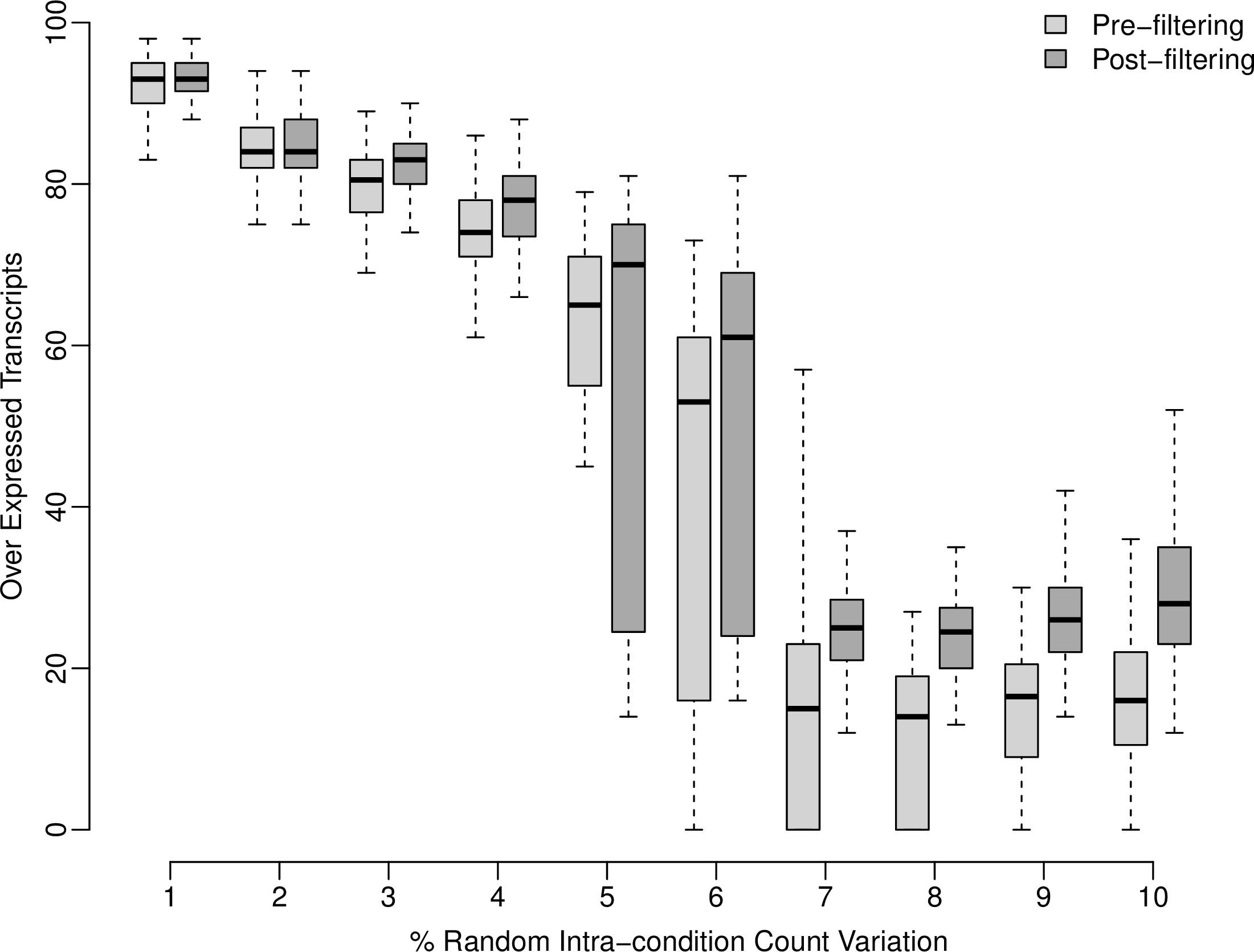

Supplement: S5 Fig — The number of transcripts identified by DESeq2 as being over expressed both prior to (light gray) and post (dark gray) filtering within each of the one hundred iterations performed at each level of introduced random intra-condition count variation. Each iteration involved initially simulating ten count datasets divided into conditions A and B following which DESeq2 was run to attempt to identify the one hundred transcripts selected for over representation as described in the methods. Following this the ten simulated datasets were filtered using TVScript with a 95th percentile threshold in order to generate corresponding filtered datasets (divided into corresponding conditions A’ and B’) on which DESeq2 was re-run. (TIF) [file pone.0274591.s005.tif]

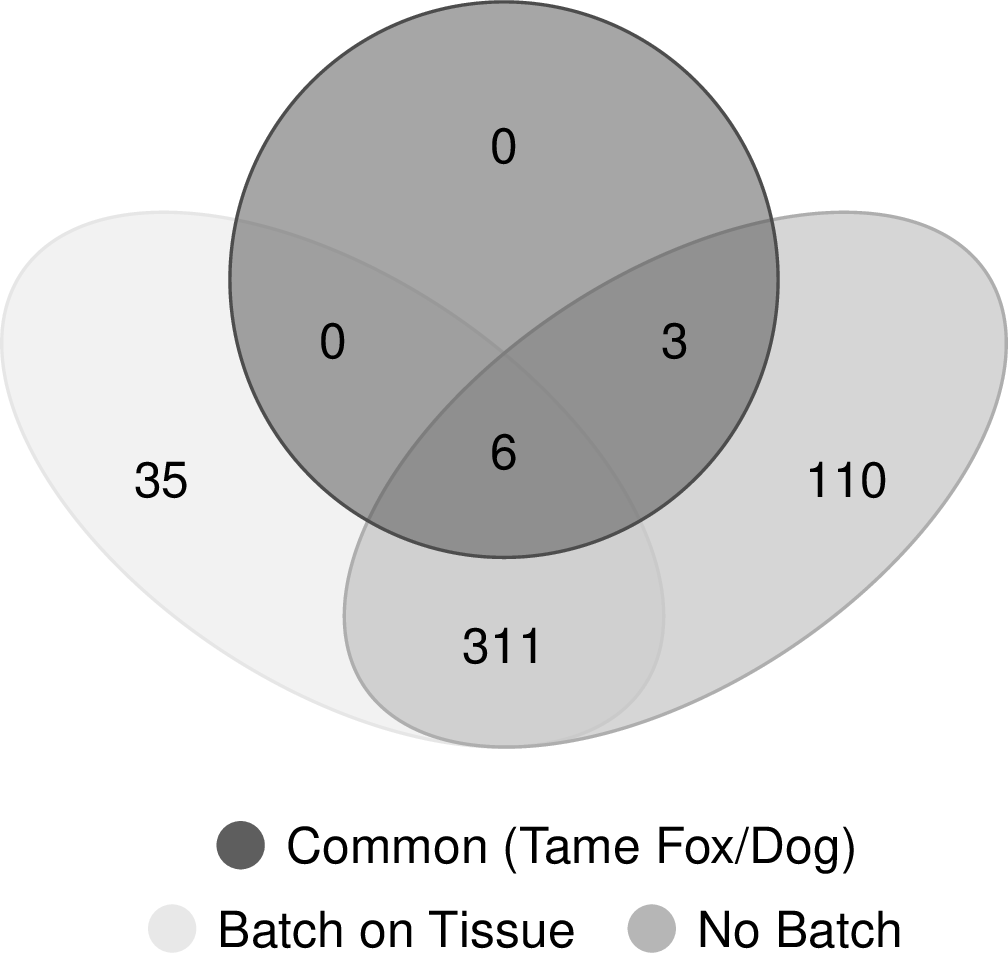

Supplement: S6 Fig — The upper dark grey circle contains the nine genes identified as being either commonly over, or under, expressed simultaniously within dogs and tame foxes using filter levels the 95th and 97th percentiles whilst only accounting for condition (wolves vs. dogs and aggressive vs. tame fox). Six of these genes (RGR, CHRNA5, MYO7A, TRIB2, STMND1 and OASL) are present when DESeq2 is run whilst also accounting for differences in tissue (light grey left oval). SQLE, ARHGAP25 and ITGA7 are observed only within the differentially expressed transcript list that is based solely on condition (dark grey right oval). (TIF) [file pone.0274591.s006.tif]

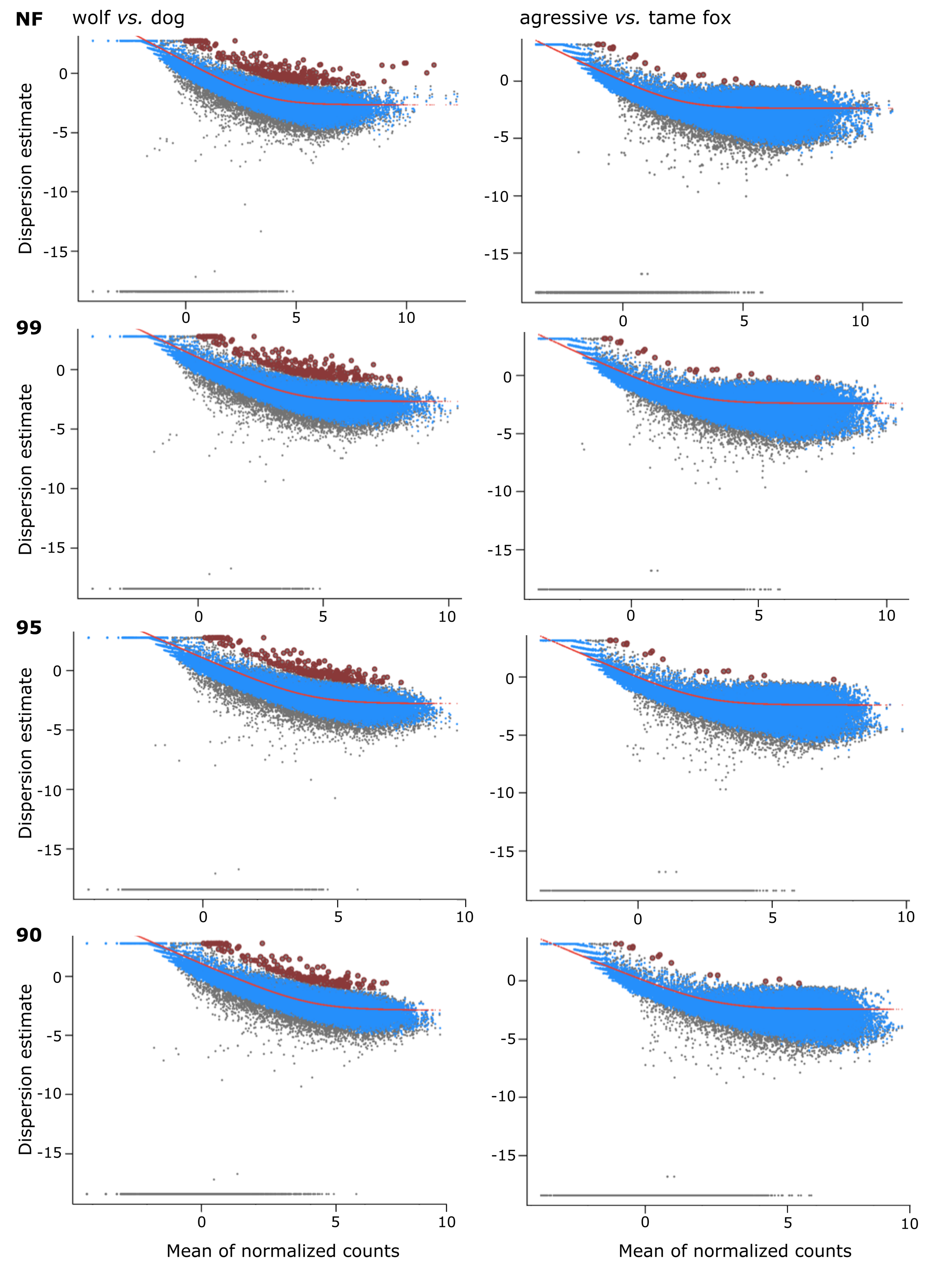

Supplement: S7 Fig — Plots of dispersion estimates in relation to the mean of normalized counts for both case studies, wolves and dogs (left panels), and tame and aggressive foxes (right panels). Estimates were calculated using DESeq2 for the non-filtered (NF) and all filtered datasets (99th, 95th and 90th are shown as an example). Gray dots represent the gene-wise maximum likelihood estimates (MLE), the red curve shows the fit to the MLEs, and blue dots identify the final maximum a posteriori (MAP) estimates of dispersion. Red dots represent the outliers detected by DESeq2. Both x and y-axis are transformed into a logarithm scale. (TIF) [file pone.0274591.s007.tif]
