# Supplementary material for: On taming the effect of transcript level intra-condition count variation during differential expression analysis: A story of dogs, foxes and wolves
Source: PLoS One. 2022 Sep 22;17(9):e0274591. doi: 10.1371/journal.pone.0274591 (PMC9498955; doi:10.1371/journal.pone.0274591)
Supplement: S2 Table — The number of transcripts from the dog reference set that are commonly identified by DESeq2 as being over expressed within condition B both prior to and post filtering for each of the one hundred iterations performed at each level of introduced random intra-condition count variation. Each iteration involved simulating ten count datasets divided into conditions A and B following which DESeq2 was run to attempt to identify the one hundred transcripts selected for over representation as described in the methods section. Filtering involved running TVScript with a 95th percentile threshold on the non-filtered datasets to generate corresponding filtered datasets (divided into corresponding conditions A’ and B’) following which DESeq2 was re-run and the results compared back to those obtained for the non filtered data. (DOCX) [file pone.0274591.s009.docx]

|  | **% Randomly Selected for Over Representation** | | | | | | | | | |
| --- | --- | --- | --- | --- | --- | --- | --- | --- | --- | --- |
| **Iteration** | **1** | **2** | **3** | **4** | **5** | **6** | **7** | **8** | **9** | **10** |
| **rep_0** | 95 | 86 | 75 | 59 | 3 | 0 | 0 | 2 | 6 | 8 |
| **rep_1** | 97 | 85 | 79 | 66 | 70 | 0 | 0 | 13 | 5 | 6 |
| **rep_2** | 85 | 86 | 67 | 70 | 41 | 22 | 21 | 0 | 14 | 9 |
| **rep_3** | 83 | 85 | 78 | 60 | 20 | 32 | 0 | 12 | 9 | 5 |
| **rep_4** | 94 | 88 | 66 | 69 | 58 | 7 | 7 | 8 | 18 | 0 |
| **rep_5** | 91 | 84 | 71 | 74 | 6 | 14 | 19 | 9 | 14 | 12 |
| **rep_6** | 92 | 86 | 77 | 58 | 62 | 13 | 15 | 13 | 0 | 18 |
| **rep_7** | 91 | 80 | 75 | 19 | 34 | 37 | 0 | 0 | 2 | 0 |
| **rep_8** | 83 | 78 | 79 | 71 | 71 | 0 | 0 | 6 | 4 | 10 |
| **rep_9** | 85 | 83 | 79 | 68 | 51 | 0 | 0 | 9 | 3 | 10 |
| **rep_10** | 88 | 89 | 78 | 66 | 63 | 37 | 0 | 12 | 2 | 0 |
| **rep_11** | 85 | 87 | 72 | 69 | 46 | 42 | 18 | 4 | 2 | 0 |
| **rep_12** | 90 | 80 | 71 | 69 | 69 | 23 | 11 | 14 | 16 | 11 |
| **rep_13** | 94 | 85 | 83 | 17 | 16 | 2 | 0 | 11 | 4 | 11 |
| **rep_14** | 81 | 84 | 86 | 71 | 14 | 15 | 0 | 11 | 12 | 11 |
| **rep_15** | 78 | 85 | 74 | 66 | 67 | 0 | 14 | 0 | 8 | 8 |
| **rep_16** | 92 | 89 | 80 | 67 | 16 | 19 | 17 | 4 | 10 | 0 |
| **rep_17** | 89 | 77 | 74 | 76 | 12 | 40 | 0 | 3 | 9 | 14 |
| **rep_18** | 93 | 84 | 80 | 46 | 58 | 16 | 7 | 4 | 15 | 4 |
| **rep_19** | 90 | 88 | 74 | 19 | 0 | 11 | 0 | 9 | 0 | 16 |
| **rep_20** | 90 | 84 | 76 | 63 | 14 | 0 | 0 | 0 | 2 | 10 |
| **rep_21** | 89 | 84 | 78 | 68 | 18 | 33 | 0 | 12 | 10 | 11 |
| **rep_22** | 81 | 80 | 72 | 71 | 51 | 0 | 0 | 11 | 0 | 14 |
| **rep_23** | 86 | 85 | 80 | 71 | 68 | 5 | 10 | 2 | 9 | 11 |
| **rep_24** | 86 | 81 | 84 | 18 | 37 | 1 | 0 | 0 | 0 | 9 |
| **rep_25** | 91 | 85 | 66 | 66 | 55 | 40 | 0 | 9 | 13 | 0 |
| **rep_26** | 91 | 89 | 74 | 69 | 61 | 0 | 9 | 11 | 0 | 10 |
| **rep_27** | 87 | 80 | 72 | 71 | 43 | 54 | 0 | 0 | 11 | 2 |
| **rep_28** | 89 | 89 | 69 | 67 | 63 | 17 | 6 | 13 | 7 | 4 |
| **rep_29** | 93 | 86 | 70 | 78 | 58 | 0 | 0 | 0 | 16 | 11 |
| **rep_30** | 96 | 90 | 69 | 74 | 61 | 12 | 3 | 0 | 16 | 0 |
| **rep_31** | 87 | 91 | 83 | 75 | 57 | 0 | 10 | 10 | 8 | 9 |
| **rep_32** | 79 | 79 | 76 | 77 | 13 | 0 | 10 | 0 | 4 | 9 |
| **rep_33** | 96 | 81 | 73 | 15 | 9 | 13 | 0 | 13 | 8 | 10 |
| **rep_34** | 93 | 79 | 71 | 79 | 63 | 12 | 8 | 5 | 10 | 7 |
| **rep_35** | 82 | 82 | 82 | 76 | 46 | 0 | 0 | 0 | 1 | 5 |
| **rep_36** | 91 | 81 | 79 | 79 | 17 | 0 | 0 | 13 | 11 | 0 |
| **rep_37** | 83 | 78 | 78 | 22 | 54 | 2 | 0 | 11 | 3 | 11 |
| **rep_38** | 91 | 88 | 81 | 20 | 51 | 46 | 0 | 0 | 12 | 6 |
| **rep_39** | 91 | 80 | 73 | 69 | 51 | 11 | 31 | 2 | 9 | 11 |
| **rep_40** | 86 | 87 | 72 | 54 | 34 | 20 | 0 | 16 | 5 | 0 |
| **rep_41** | 89 | 89 | 80 | 65 | 9 | 15 | 0 | 7 | 13 | 8 |
| **rep_42** | 85 | 84 | 81 | 74 | 11 | 11 | 0 | 6 | 16 | 12 |
| **rep_43** | 97 | 80 | 75 | 60 | 44 | 22 | 14 | 4 | 9 | 10 |
| **rep_44** | 79 | 87 | 74 | 56 | 54 | 0 | 17 | 12 | 0 | 1 |
| **rep_45** | 91 | 82 | 71 | 73 | 60 | 0 | 13 | 11 | 11 | 12 |
| **rep_46** | 84 | 84 | 71 | 63 | 49 | 40 | 4 | 6 | 9 | 11 |
| **rep_47** | 95 | 76 | 79 | 75 | 14 | 46 | 11 | 17 | 8 | 12 |
| **rep_48** | 86 | 87 | 67 | 57 | 19 | 0 | 9 | 15 | 13 | 10 |
| **rep_49** | 92 | 83 | 71 | 20 | 11 | 11 | 0 | 11 | 13 | 8 |
| **rep_50** | 84 | 79 | 82 | 75 | 9 | 12 | 10 | 3 | 6 | 10 |
| **rep_51** | 92 | 77 | 74 | 18 | 9 | 20 | 0 | 8 | 9 | 5 |
| **rep_52** | 93 | 83 | 83 | 63 | 46 | 9 | 8 | 0 | 8 | 3 |
| **rep_53** | 90 | 83 | 75 | 64 | 4 | 0 | 9 | 0 | 7 | 7 |
| **rep_54** | 96 | 79 | 72 | 69 | 55 | 0 | 0 | 0 | 1 | 8 |
| **rep_55** | 91 | 88 | 84 | 71 | 61 | 54 | 13 | 7 | 10 | 11 |
| **rep_56** | 76 | 87 | 74 | 58 | 17 | 50 | 7 | 14 | 3 | 0 |
| **rep_57** | 84 | 87 | 71 | 47 | 56 | 0 | 0 | 8 | 11 | 12 |
| **rep_58** | 84 | 84 | 80 | 61 | 21 | 62 | 0 | 4 | 8 | 9 |
| **rep_59** | 88 | 87 | 83 | 65 | 11 | 0 | 0 | 0 | 7 | 11 |
| **rep_60** | 81 | 81 | 80 | 58 | 63 | 28 | 2 | 7 | 5 | 6 |
| **rep_61** | 80 | 82 | 81 | 64 | 21 | 0 | 3 | 7 | 12 | 0 |
| **rep_62** | 86 | 90 | 82 | 15 | 0 | 0 | 0 | 9 | 6 | 14 |
| **rep_63** | 83 | 85 | 79 | 70 | 15 | 4 | 8 | 7 | 9 | 9 |
| **rep_64** | 92 | 87 | 70 | 60 | 64 | 11 | 16 | 8 | 5 | 7 |
| **rep_65** | 88 | 80 | 85 | 17 | 39 | 14 | 9 | 7 | 11 | 10 |
| **rep_66** | 87 | 86 | 70 | 76 | 14 | 14 | 0 | 1 | 8 | 8 |
| **rep_67** | 91 | 86 | 75 | 65 | 63 | 4 | 9 | 11 | 4 | 12 |
| **rep_68** | 91 | 86 | 73 | 60 | 52 | 2 | 10 | 16 | 0 | 6 |
| **rep_69** | 88 | 81 | 78 | 63 | 55 | 7 | 0 | 6 | 10 | 4 |
| **rep_70** | 92 | 80 | 67 | 61 | 50 | 11 | 6 | 9 | 7 | 4 |
| **rep_71** | 73 | 84 | 71 | 72 | 49 | 14 | 0 | 13 | 5 | 9 |
| **rep_72** | 89 | 86 | 79 | 75 | 61 | 0 | 4 | 5 | 9 | 13 |
| **rep_73** | 94 | 80 | 76 | 78 | 50 | 13 | 0 | 11 | 13 | 14 |
| **rep_74** | 87 | 75 | 71 | 52 | 45 | 13 | 11 | 6 | 0 | 8 |
| **rep_75** | 88 | 85 | 72 | 62 | 67 | 48 | 15 | 2 | 6 | 10 |
| **rep_76** | 95 | 86 | 74 | 67 | 54 | 0 | 8 | 0 | 0 | 13 |
| **rep_77** | 82 | 81 | 64 | 72 | 16 | 0 | 9 | 12 | 9 | 6 |
| **rep_78** | 94 | 79 | 77 | 47 | 15 | 0 | 10 | 11 | 7 | 12 |
| **rep_79** | 86 | 82 | 76 | 17 | 55 | 35 | 0 | 4 | 10 | 0 |
| **rep_80** | 86 | 76 | 77 | 63 | 11 | 0 | 0 | 8 | 12 | 14 |
| **rep_81** | 94 | 85 | 85 | 54 | 65 | 31 | 4 | 0 | 10 | 14 |
| **rep_82** | 87 | 87 | 75 | 55 | 53 | 14 | 0 | 12 | 10 | 6 |
| **rep_83** | 96 | 81 | 67 | 76 | 4 | 38 | 5 | 5 | 6 | 8 |
| **rep_84** | 81 | 84 | 77 | 66 | 66 | 12 | 12 | 0 | 0 | 3 |
| **rep_85** | 77 | 82 | 83 | 72 | 15 | 48 | 0 | 6 | 11 | 15 |
| **rep_86** | 88 | 89 | 65 | 72 | 57 | 14 | 0 | 8 | 2 | 7 |
| **rep_87** | 86 | 83 | 84 | 69 | 59 | 16 | 12 | 9 | 11 | 8 |
| **rep_88** | 91 | 84 | 77 | 60 | 45 | 38 | 0 | 16 | 2 | 13 |
| **rep_89** | 91 | 86 | 75 | 54 | 11 | 0 | 8 | 10 | 9 | 8 |
| **rep_90** | 92 | 87 | 73 | 62 | 18 | 49 | 3 | 0 | 12 | 12 |
| **rep_91** | 89 | 84 | 81 | 19 | 53 | 0 | 20 | 7 | 0 | 11 |
| **rep_92** | 90 | 83 | 72 | 68 | 15 | 36 | 0 | 15 | 6 | 6 |
| **rep_93** | 92 | 78 | 74 | 63 | 38 | 55 | 16 | 0 | 5 | 0 |
| **rep_94** | 77 | 83 | 83 | 56 | 2 | 0 | 16 | 7 | 9 | 2 |
| **rep_95** | 85 | 87 | 78 | 66 | 16 | 50 | 14 | 10 | 7 | 7 |
| **rep_96** | 89 | 86 | 74 | 79 | 53 | 45 | 5 | 9 | 0 | 10 |
| **rep_97** | 90 | 83 | 70 | 14 | 64 | 30 | 0 | 5 | 6 | 6 |
| **rep_98** | 90 | 80 | 72 | 17 | 47 | 2 | 0 | 13 | 7 | 12 |
| **rep_99** | 90 | 82 | 85 | 63 | 63 | 33 | 17 | 0 | 9 | 14 |
