# Supplementary material for: On taming the effect of transcript level intra-condition count variation during differential expression analysis: A story of dogs, foxes and wolves
Source: PLoS One. 2022 Sep 22;17(9):e0274591. doi: 10.1371/journal.pone.0274591 (PMC9498955; doi:10.1371/journal.pone.0274591)
Supplement: S3 Table — Same as S2 Table but where the one hundred transcripts selected for over representation within condition B are re-selected during each iteration. (DOCX) [file pone.0274591.s010.docx]

|  | **% Randomly Selected for Over Representation** | | | | | | | | | |
| --- | --- | --- | --- | --- | --- | --- | --- | --- | --- | --- |
| **Iteration** | **1** | **2** | **3** | **4** | **5** | **6** | **7** | **8** | **9** | **10** |
| **rep_0** | 91 | 73 | 78 | 65 | 46 | 0 | 17 | 11 | 11 | 8 |
| **rep_1** | 85 | 68 | 80 | 69 | 55 | 12 | 0 | 0 | 6 | 0 |
| **rep_2** | 96 | 83 | 68 | 53 | 14 | 13 | 0 | 7 | 9 | 14 |
| **rep_3** | 92 | 87 | 77 | 70 | 14 | 13 | 4 | 14 | 4 | 7 |
| **rep_4** | 90 | 75 | 74 | 72 | 63 | 56 | 13 | 0 | 6 | 0 |
| **rep_5** | 93 | 81 | 73 | 25 | 55 | 43 | 13 | 0 | 0 | 4 |
| **rep_6** | 89 | 94 | 74 | 75 | 15 | 11 | 16 | 0 | 3 | 9 |
| **rep_7** | 92 | 79 | 74 | 13 | 67 | 7 | 0 | 9 | 9 | 8 |
| **rep_8** | 93 | 79 | 74 | 66 | 67 | 0 | 7 | 11 | 6 | 9 |
| **rep_9** | 90 | 83 | 80 | 69 | 58 | 2 | 0 | 4 | 10 | 6 |
| **rep_10** | 89 | 77 | 74 | 59 | 12 | 0 | 0 | 0 | 1 | 14 |
| **rep_11** | 74 | 79 | 86 | 16 | 48 | 0 | 13 | 3 | 10 | 9 |
| **rep_12** | 94 | 81 | 72 | 65 | 49 | 14 | 0 | 14 | 9 | 5 |
| **rep_13** | 83 | 87 | 85 | 19 | 7 | 26 | 0 | 9 | 7 | 3 |
| **rep_14** | 73 | 70 | 74 | 60 | 56 | 47 | 1 | 8 | 8 | 2 |
| **rep_15** | 90 | 78 | 82 | 79 | 61 | 16 | 0 | 0 | 8 | 8 |
| **rep_16** | 92 | 81 | 80 | 63 | 60 | 14 | 0 | 0 | 11 | 10 |
| **rep_17** | 92 | 81 | 78 | 66 | 26 | 16 | 14 | 6 | 9 | 12 |
| **rep_18** | 83 | 86 | 72 | 58 | 57 | 15 | 15 | 12 | 0 | 2 |
| **rep_19** | 92 | 83 | 72 | 65 | 58 | 32 | 0 | 7 | 1 | 0 |
| **rep_20** | 82 | 83 | 81 | 60 | 62 | 40 | 5 | 0 | 4 | 6 |
| **rep_21** | 92 | 79 | 67 | 74 | 15 | 10 | 0 | 12 | 2 | 10 |
| **rep_22** | 92 | 88 | 77 | 64 | 26 | 0 | 13 | 12 | 8 | 0 |
| **rep_23** | 85 | 87 | 72 | 63 | 14 | 46 | 13 | 15 | 11 | 5 |
| **rep_24** | 79 | 78 | 78 | 67 | 7 | 50 | 0 | 10 | 7 | 0 |
| **rep_25** | 77 | 81 | 78 | 14 | 21 | 15 | 10 | 9 | 2 | 8 |
| **rep_26** | 80 | 83 | 74 | 73 | 52 | 15 | 13 | 5 | 0 | 6 |
| **rep_27** | 98 | 81 | 81 | 73 | 3 | 24 | 15 | 0 | 16 | 2 |
| **rep_28** | 89 | 88 | 72 | 76 | 59 | 15 | 5 | 3 | 0 | 0 |
| **rep_29** | 90 | 86 | 75 | 11 | 45 | 14 | 7 | 11 | 16 | 0 |
| **rep_30** | 83 | 84 | 73 | 62 | 0 | 12 | 17 | 7 | 8 | 8 |
| **rep_31** | 82 | 86 | 70 | 58 | 13 | 21 | 8 | 5 | 9 | 4 |
| **rep_32** | 93 | 88 | 76 | 63 | 18 | 0 | 0 | 4 | 0 | 0 |
| **rep_33** | 92 | 81 | 74 | 61 | 2 | 54 | 0 | 8 | 0 | 1 |
| **rep_34** | 90 | 84 | 76 | 69 | 8 | 13 | 14 | 3 | 2 | 5 |
| **rep_35** | 83 | 70 | 77 | 72 | 53 | 34 | 0 | 1 | 11 | 5 |
| **rep_36** | 82 | 90 | 88 | 69 | 64 | 16 | 3 | 8 | 8 | 10 |
| **rep_37** | 84 | 83 | 74 | 59 | 12 | 16 | 0 | 0 | 4 | 2 |
| **rep_38** | 92 | 86 | 73 | 78 | 11 | 14 | 10 | 6 | 9 | 4 |
| **rep_39** | 92 | 83 | 80 | 68 | 64 | 41 | 0 | 0 | 13 | 3 |
| **rep_40** | 91 | 81 | 71 | 77 | 50 | 17 | 10 | 18 | 4 | 5 |
| **rep_41** | 96 | 83 | 71 | 63 | 14 | 5 | 0 | 0 | 12 | 1 |
| **rep_42** | 94 | 87 | 69 | 54 | 15 | 17 | 0 | 19 | 8 | 8 |
| **rep_43** | 95 | 87 | 68 | 53 | 39 | 0 | 10 | 2 | 0 | 4 |
| **rep_44** | 84 | 85 | 76 | 22 | 39 | 54 | 3 | 0 | 9 | 5 |
| **rep_45** | 91 | 74 | 62 | 71 | 36 | 0 | 11 | 10 | 2 | 12 |
| **rep_46** | 94 | 69 | 80 | 61 | 12 | 9 | 7 | 14 | 12 | 5 |
| **rep_47** | 91 | 86 | 72 | 71 | 14 | 44 | 0 | 1 | 0 | 1 |
| **rep_48** | 92 | 83 | 77 | 67 | 40 | 37 | 0 | 9 | 8 | 3 |
| **rep_49** | 79 | 78 | 72 | 79 | 9 | 8 | 11 | 16 | 0 | 5 |
| **rep_50** | 88 | 87 | 66 | 11 | 12 | 35 | 0 | 6 | 11 | 4 |
| **rep_51** | 88 | 89 | 82 | 76 | 10 | 9 | 0 | 8 | 5 | 0 |
| **rep_52** | 91 | 89 | 79 | 67 | 6 | 6 | 11 | 7 | 13 | 7 |
| **rep_53** | 97 | 85 | 76 | 63 | 0 | 8 | 13 | 10 | 0 | 2 |
| **rep_54** | 89 | 83 | 61 | 56 | 49 | 17 | 9 | 5 | 0 | 0 |
| **rep_55** | 90 | 86 | 80 | 62 | 10 | 12 | 17 | 3 | 0 | 0 |
| **rep_56** | 91 | 83 | 16 | 70 | 3 | 8 | 12 | 0 | 0 | 11 |
| **rep_57** | 96 | 82 | 78 | 79 | 36 | 0 | 2 | 12 | 15 | 0 |
| **rep_58** | 95 | 83 | 77 | 64 | 57 | 47 | 0 | 6 | 3 | 3 |
| **rep_59** | 93 | 76 | 82 | 53 | 45 | 0 | 0 | 15 | 3 | 12 |
| **rep_60** | 94 | 83 | 78 | 65 | 1 | 37 | 10 | 6 | 7 | 8 |
| **rep_61** | 81 | 82 | 74 | 18 | 37 | 8 | 0 | 11 | 8 | 0 |
| **rep_62** | 76 | 80 | 79 | 61 | 73 | 0 | 5 | 4 | 7 | 21 |
| **rep_63** | 95 | 80 | 55 | 74 | 59 | 17 | 42 | 0 | 8 | 13 |
| **rep_64** | 91 | 83 | 75 | 69 | 0 | 4 | 47 | 0 | 7 | 5 |
| **rep_65** | 92 | 78 | 72 | 58 | 17 | 59 | 0 | 5 | 0 | 0 |
| **rep_66** | 88 | 85 | 72 | 69 | 47 | 14 | 9 | 5 | 0 | 13 |
| **rep_67** | 96 | 93 | 60 | 63 | 43 | 0 | 7 | 0 | 7 | 5 |
| **rep_68** | 89 | 82 | 73 | 62 | 59 | 11 | 10 | 0 | 11 | 3 |
| **rep_69** | 91 | 79 | 77 | 68 | 55 | 0 | 0 | 9 | 7 | 11 |
| **rep_70** | 93 | 82 | 83 | 69 | 14 | 39 | 9 | 0 | 7 | 11 |
| **rep_71** | 93 | 82 | 79 | 69 | 16 | 0 | 2 | 0 | 9 | 2 |
| **rep_72** | 81 | 81 | 69 | 80 | 0 | 54 | 40 | 0 | 1 | 0 |
| **rep_73** | 87 | 80 | 70 | 75 | 52 | 48 | 0 | 0 | 2 | 4 |
| **rep_74** | 88 | 81 | 75 | 68 | 65 | 38 | 5 | 0 | 10 | 3 |
| **rep_75** | 83 | 82 | 80 | 66 | 25 | 0 | 10 | 0 | 5 | 4 |
| **rep_76** | 92 | 80 | 82 | 63 | 56 | 37 | 0 | 0 | 0 | 2 |
| **rep_77** | 89 | 84 | 82 | 21 | 1 | 9 | 10 | 4 | 11 | 6 |
| **rep_78** | 89 | 77 | 78 | 19 | 12 | 0 | 9 | 0 | 8 | 0 |
| **rep_79** | 74 | 75 | 79 | 72 | 57 | 15 | 1 | 12 | 9 | 5 |
| **rep_80** | 90 | 89 | 82 | 18 | 53 | 53 | 18 | 15 | 11 | 0 |
| **rep_81** | 98 | 88 | 88 | 65 | 12 | 51 | 11 | 0 | 5 | 12 |
| **rep_82** | 91 | 81 | 78 | 67 | 13 | 59 | 0 | 0 | 1 | 0 |
| **rep_83** | 93 | 88 | 80 | 68 | 17 | 17 | 4 | 12 | 0 | 3 |
| **rep_84** | 88 | 80 | 69 | 58 | 38 | 46 | 7 | 14 | 4 | 9 |
| **rep_85** | 94 | 83 | 68 | 64 | 54 | 8 | 13 | 13 | 10 | 14 |
| **rep_86** | 94 | 85 | 76 | 63 | 66 | 60 | 0 | 0 | 0 | 12 |
| **rep_87** | 94 | 74 | 80 | 69 | 21 | 5 | 0 | 17 | 10 | 0 |
| **rep_88** | 94 | 82 | 65 | 51 | 52 | 0 | 0 | 4 | 9 | 7 |
| **rep_89** | 82 | 83 | 82 | 79 | 64 | 0 | 5 | 7 | 0 | 1 |
| **rep_90** | 92 | 88 | 82 | 66 | 9 | 50 | 0 | 13 | 3 | 6 |
| **rep_91** | 89 | 86 | 61 | 67 | 42 | 13 | 7 | 0 | 9 | 11 |
| **rep_92** | 97 | 79 | 82 | 67 | 57 | 0 | 3 | 0 | 5 | 3 |
| **rep_93** | 77 | 76 | 68 | 80 | 30 | 26 | 2 | 5 | 0 | 1 |
| **rep_94** | 93 | 82 | 65 | 13 | 77 | 9 | 0 | 0 | 4 | 9 |
| **rep_95** | 89 | 76 | 80 | 60 | 60 | 0 | 12 | 11 | 6 | 9 |
| **rep_96** | 72 | 78 | 69 | 74 | 17 | 0 | 0 | 1 | 7 | 3 |
| **rep_97** | 91 | 81 | 74 | 65 | 68 | 39 | 14 | 6 | 2 | 3 |
| **rep_98** | 82 | 85 | 78 | 71 | 53 | 14 | 5 | 6 | 5 | 0 |
| **rep_99** | 75 | 79 | 78 | 70 | 60 | 46 | 9 | 4 | 6 | 3 |
