# Supplementary material for: On taming the effect of transcript level intra-condition count variation during differential expression analysis: A story of dogs, foxes and wolves
Source: PLoS One. 2022 Sep 22;17(9):e0274591. doi: 10.1371/journal.pone.0274591 (PMC9498955; doi:10.1371/journal.pone.0274591)
Supplement: S5 Table — Numbers in S3 Table were divided by the maximum number of over expressed transcripts detected within each correspomding iteration i.e. the maximum number detected using corresponding non-filtered and filtered datasets. (DOCX) [file pone.0274591.s012.docx]

|  | **% Randomly Selected for Over Representation** | | | | | | | | | |
| --- | --- | --- | --- | --- | --- | --- | --- | --- | --- | --- |
| **Iteration** | **1** | **2** | **3** | **4** | **5** | **6** | **7** | **8** | **9** | **10** |
| **rep_0** | 0.99 | 0.90 | 0.92 | 0.90 | 0.75 | 0.00 | 0.68 | 0.58 | 0.65 | 0.53 |
| **rep_1** | 0.96 | 0.89 | 0.98 | 0.92 | 0.75 | 0.20 | 0.00 | NA | 0.38 | NA |
| **rep_2** | 0.99 | 1.00 | 0.93 | 0.84 | 0.74 | 0.19 | NA | 0.37 | 0.41 | 0.61 |
| **rep_3** | 0.99 | 1.00 | 0.96 | 0.89 | 0.23 | 0.23 | 0.40 | 0.64 | 0.29 | 0.58 |
| **rep_4** | 0.97 | 0.95 | 0.93 | 0.90 | 0.83 | 0.82 | 0.62 | NA | 0.50 | 0.00 |
| **rep_5** | 0.99 | 0.99 | 0.94 | 0.86 | 0.85 | 0.72 | 0.65 | NA | NA | 0.40 |
| **rep_6** | 1.00 | 1.00 | 0.97 | 0.93 | 0.20 | 0.44 | 0.64 | 0.00 | 0.27 | 0.41 |
| **rep_7** | 0.99 | 0.99 | 0.97 | 0.17 | 0.93 | 0.30 | NA | 0.36 | 0.47 | 0.42 |
| **rep_8** | 0.98 | 0.99 | 0.91 | 0.93 | 0.92 | NA | 0.39 | 0.58 | 0.40 | 0.50 |
| **rep_9** | 0.99 | 0.97 | 0.95 | 0.88 | 0.82 | 0.09 | NA | 0.33 | 0.43 | 0.22 |
| **rep_10** | 0.98 | 0.99 | 0.91 | 0.88 | 0.18 | NA | 0.00 | 0.00 | 0.33 | 0.58 |
| **rep_11** | 0.88 | 0.93 | 1.00 | 0.21 | 0.80 | NA | 0.62 | 0.27 | 0.50 | 0.41 |
| **rep_12** | 0.98 | 0.99 | 0.99 | 0.92 | 0.79 | 0.20 | NA | 0.74 | 0.45 | 0.33 |
| **rep_13** | 0.92 | 0.99 | 0.98 | 0.27 | 0.11 | 0.59 | NA | 0.41 | 0.33 | 0.21 |
| **rep_14** | 0.88 | 0.85 | 0.97 | 0.90 | 0.89 | 0.78 | 0.08 | 0.50 | 0.44 | 0.15 |
| **rep_15** | 0.96 | 1.00 | 0.99 | 0.96 | 0.86 | 0.29 | NA | NA | 0.40 | 0.47 |
| **rep_16** | 1.00 | 1.00 | 0.98 | 0.88 | 0.83 | 0.25 | 0.00 | NA | 0.50 | 0.53 |
| **rep_17** | 0.96 | 0.99 | 0.96 | 0.88 | 0.57 | 0.62 | 0.61 | 0.38 | 0.43 | 0.44 |
| **rep_18** | 0.91 | 0.98 | 0.90 | 0.91 | 0.85 | 0.25 | 0.68 | 0.52 | 0.00 | 0.13 |
| **rep_19** | 0.97 | 0.98 | 0.88 | 0.84 | 0.87 | 0.65 | NA | 0.54 | 0.13 | 0.00 |
| **rep_20** | 0.91 | 0.99 | 0.94 | 0.88 | 0.93 | 0.70 | 0.31 | NA | 0.31 | 0.33 |
| **rep_21** | 0.97 | 0.92 | 0.93 | 0.96 | 0.21 | 0.17 | 0.00 | 0.67 | 0.18 | 0.40 |
| **rep_22** | 0.96 | 1.00 | 0.92 | 0.90 | 0.37 | NA | 0.22 | 0.50 | 0.40 | NA |
| **rep_23** | 0.94 | 0.99 | 0.96 | 0.86 | 0.20 | 0.75 | 0.59 | 0.79 | 0.65 | 0.23 |
| **rep_24** | 0.91 | 0.95 | 0.94 | 0.94 | 0.58 | 0.79 | NA | 0.56 | 0.44 | 0.00 |
| **rep_25** | 0.90 | 0.96 | 0.94 | 0.20 | 0.33 | 0.22 | 0.45 | 0.64 | 0.18 | 0.36 |
| **rep_26** | 0.93 | 0.98 | 0.93 | 0.92 | 0.81 | 0.29 | 0.22 | 0.38 | NA | 0.40 |
| **rep_27** | 1.00 | 0.98 | 0.94 | 0.95 | 0.15 | 0.86 | 0.75 | NA | 0.62 | 0.25 |
| **rep_28** | 0.97 | 1.00 | 0.92 | 0.95 | 0.81 | 0.28 | 0.38 | 0.30 | NA | 0.00 |
| **rep_29** | 0.97 | 1.00 | 0.96 | 0.17 | 0.76 | 0.21 | 0.35 | 0.50 | 0.59 | 0.00 |
| **rep_30** | 0.93 | 1.00 | 0.92 | 0.86 | NA | 0.22 | 0.65 | 0.58 | 0.36 | 0.42 |
| **rep_31** | 0.92 | 0.98 | 0.93 | 0.85 | 0.18 | 0.47 | 0.47 | 0.50 | 0.35 | 0.21 |
| **rep_32** | 1.00 | 0.98 | 0.95 | 0.88 | 0.25 | NA | NA | 0.31 | 0.00 | 0.00 |
| **rep_33** | 0.98 | 0.95 | 0.96 | 0.91 | 0.08 | 0.82 | NA | 0.50 | 0.00 | 0.08 |
| **rep_34** | 0.96 | 1.00 | 0.97 | 0.91 | 0.26 | 0.21 | 0.58 | 0.43 | 0.17 | 0.29 |
| **rep_35** | 0.92 | 0.95 | 0.94 | 0.92 | 0.79 | 0.71 | NA | 0.13 | 0.52 | 0.36 |
| **rep_36** | 0.87 | 1.00 | 0.99 | 0.93 | 0.91 | 0.62 | 0.27 | 0.33 | 0.47 | 0.56 |
| **rep_37** | 0.93 | 0.98 | 0.95 | 0.86 | 0.18 | 0.24 | NA | NA | 0.29 | 0.15 |
| **rep_38** | 0.96 | 0.99 | 0.89 | 0.95 | 0.18 | 0.22 | 0.40 | 0.32 | 0.45 | 0.25 |
| **rep_39** | 0.97 | 0.94 | 0.96 | 0.92 | 0.85 | 0.71 | NA | NA | 0.43 | 0.23 |
| **rep_40** | 0.98 | 0.95 | 0.93 | 0.96 | 0.79 | 0.27 | 0.17 | 0.72 | 0.50 | 0.31 |
| **rep_41** | 0.99 | 0.99 | 0.95 | 0.82 | 0.19 | 0.22 | NA | NA | 0.52 | 0.17 |
| **rep_42** | 0.99 | 0.99 | 0.95 | 0.78 | 0.21 | 0.68 | NA | 0.73 | 0.33 | 0.32 |
| **rep_43** | 0.97 | 0.98 | 0.89 | 0.85 | 0.71 | NA | 0.53 | 0.22 | NA | 0.22 |
| **rep_44** | 0.93 | 0.98 | 0.95 | 0.28 | 0.70 | 0.86 | 0.19 | NA | 0.36 | 0.26 |
| **rep_45** | 0.98 | 0.96 | 0.89 | 0.89 | 0.65 | NA | 0.58 | 0.59 | 0.17 | 0.55 |
| **rep_46** | 1.00 | 0.87 | 0.94 | 0.87 | 0.15 | 0.29 | 0.44 | 0.58 | 0.48 | 0.31 |
| **rep_47** | 0.99 | 0.99 | 0.90 | 0.95 | 0.67 | 0.69 | NA | 0.06 | NA | 0.08 |
| **rep_48** | 0.98 | 0.99 | 0.92 | 0.92 | 0.74 | 0.66 | NA | 0.50 | 0.44 | 0.27 |
| **rep_49** | 0.92 | 0.98 | 0.95 | 0.94 | 0.38 | 0.53 | 0.20 | 0.59 | NA | 0.31 |
| **rep_50** | 0.98 | 0.99 | 0.89 | 0.20 | 0.17 | 0.64 | NA | 0.43 | 0.48 | 0.14 |
| **rep_51** | 0.99 | 0.99 | 0.98 | 0.94 | 0.20 | 0.17 | NA | 0.40 | 0.29 | 0.00 |
| **rep_52** | 0.98 | 0.98 | 0.95 | 0.94 | 0.11 | 0.16 | 0.19 | 0.41 | 0.76 | 0.39 |
| **rep_53** | 1.00 | 0.98 | 0.97 | 0.89 | NA | 0.50 | 0.57 | 0.48 | 0.00 | 0.17 |
| **rep_54** | 0.97 | 0.98 | 0.88 | 0.82 | 0.75 | 0.71 | 0.47 | 0.33 | 0.00 | NA |
| **rep_55** | 0.98 | 0.97 | 0.95 | 0.86 | 0.15 | 0.23 | 0.28 | 0.20 | NA | NA |
| **rep_56** | 0.98 | 0.98 | 0.25 | 0.91 | 0.14 | 0.15 | 0.46 | 0.00 | NA | 0.50 |
| **rep_57** | 0.99 | 0.93 | 0.95 | 0.95 | 0.65 | NA | 0.29 | 0.60 | 0.60 | 0.00 |
| **rep_58** | 0.98 | 0.99 | 0.92 | 0.89 | 0.81 | 0.73 | NA | 0.40 | 0.18 | 0.20 |
| **rep_59** | 0.98 | 0.97 | 0.96 | 0.87 | 0.74 | NA | NA | 0.71 | 0.21 | 0.39 |
| **rep_60** | 0.98 | 0.98 | 0.94 | 0.88 | 0.05 | 0.67 | 0.19 | 0.29 | 0.30 | 0.44 |
| **rep_61** | 0.91 | 0.94 | 0.93 | 0.23 | 0.67 | 0.15 | NA | 0.48 | 0.73 | 0.00 |
| **rep_62** | 0.90 | 0.98 | 0.95 | 0.86 | 0.92 | NA | 0.36 | 0.31 | 0.35 | 0.58 |
| **rep_63** | 0.99 | 0.99 | 0.89 | 0.93 | 0.86 | 0.35 | 0.70 | NA | 0.47 | 0.48 |
| **rep_64** | 0.99 | 0.99 | 0.95 | 0.93 | NA | 0.33 | 0.77 | NA | 0.35 | 0.36 |
| **rep_65** | 0.99 | 0.98 | 0.89 | 0.82 | 0.26 | 0.82 | NA | 0.38 | NA | NA |
| **rep_66** | 0.95 | 0.96 | 0.91 | 0.93 | 0.78 | 0.24 | 0.53 | 0.42 | NA | 0.57 |
| **rep_67** | 1.00 | 0.99 | 0.85 | 0.90 | 0.73 | 0.00 | 0.18 | 0.00 | 0.44 | 0.45 |
| **rep_68** | 0.98 | 0.96 | 0.91 | 0.86 | 0.81 | 0.50 | 0.27 | NA | 0.50 | 0.14 |
| **rep_69** | 0.97 | 0.93 | 0.95 | 0.91 | 0.77 | NA | NA | 0.50 | 0.47 | 0.44 |
| **rep_70** | 0.98 | 0.96 | 0.93 | 0.90 | 0.22 | 0.72 | 0.56 | NA | 0.64 | 0.39 |
| **rep_71** | 0.99 | 0.99 | 0.96 | 0.93 | 0.25 | 0.00 | 0.15 | NA | 0.43 | 0.17 |
| **rep_72** | 0.87 | 0.99 | 0.88 | 0.99 | NA | 0.81 | 0.70 | NA | 0.11 | NA |
| **rep_73** | 0.94 | 0.98 | 0.92 | 0.93 | 0.83 | 0.79 | NA | NA | 0.29 | 0.22 |
| **rep_74** | 0.98 | 0.99 | 0.96 | 0.94 | 0.89 | 0.72 | 0.50 | NA | 0.38 | 0.20 |
| **rep_75** | 0.92 | 0.98 | 0.98 | 0.88 | 0.56 | NA | 0.53 | NA | 0.56 | 0.36 |
| **rep_76** | 0.97 | 0.99 | 0.99 | 0.85 | 0.88 | 0.73 | 0.00 | NA | NA | 0.08 |
| **rep_77** | 0.98 | 0.99 | 0.96 | 0.26 | 0.05 | 0.20 | 0.17 | 0.31 | 0.65 | 0.26 |
| **rep_78** | 0.99 | 1.00 | 0.94 | 0.23 | 0.23 | NA | 0.56 | NA | 0.38 | NA |
| **rep_79** | 0.88 | 0.99 | 0.94 | 0.92 | 0.88 | 0.27 | 0.08 | 0.75 | 0.38 | 0.29 |
| **rep_80** | 0.98 | 0.98 | 0.96 | 0.24 | 0.79 | 0.82 | 0.69 | 0.60 | 0.46 | NA |
| **rep_81** | 1.00 | 0.97 | 1.00 | 0.89 | 0.16 | 0.80 | 0.17 | NA | 0.31 | 0.48 |
| **rep_82** | 0.96 | 0.98 | 0.96 | 0.87 | 0.21 | 0.81 | NA | 0.00 | 0.17 | NA |
| **rep_83** | 0.96 | 1.00 | 0.98 | 0.91 | 0.23 | 0.25 | 0.27 | 0.57 | 0.00 | 0.38 |
| **rep_84** | 0.97 | 0.98 | 0.90 | 0.87 | 0.72 | 0.79 | 0.27 | 0.67 | 0.36 | 0.47 |
| **rep_85** | 0.99 | 0.97 | 0.94 | 0.82 | 0.78 | 0.14 | 0.54 | 0.57 | 0.36 | 0.58 |
| **rep_86** | 0.98 | 0.98 | 0.90 | 0.88 | 0.88 | 0.88 | NA | NA | 0.00 | 0.52 |
| **rep_87** | 1.00 | 0.97 | 0.94 | 0.88 | 0.36 | 0.25 | NA | 0.74 | 0.53 | 0.00 |
| **rep_88** | 0.98 | 0.99 | 0.87 | 0.85 | 0.79 | NA | NA | 0.31 | 0.53 | 0.29 |
| **rep_89** | 0.91 | 0.95 | 0.94 | 0.92 | 0.94 | NA | 0.28 | 0.37 | NA | 0.10 |
| **rep_90** | 0.98 | 0.99 | 0.98 | 0.90 | 0.16 | 0.79 | NA | 0.72 | 0.21 | 0.46 |
| **rep_91** | 0.98 | 0.98 | 0.85 | 0.89 | 0.75 | 0.24 | 0.54 | NA | 0.53 | 0.61 |
| **rep_92** | 1.00 | 0.96 | 0.98 | 0.92 | 0.85 | 0.00 | 0.25 | NA | 0.26 | 0.21 |
| **rep_93** | 0.89 | 0.90 | 0.96 | 0.94 | 0.64 | 0.58 | 0.13 | 0.29 | NA | 0.08 |
| **rep_94** | 0.97 | 1.00 | 0.90 | 0.19 | 0.99 | 0.64 | NA | NA | 0.36 | 0.35 |
| **rep_95** | 0.96 | 0.95 | 0.99 | 0.83 | 0.88 | NA | 0.20 | 0.52 | 0.50 | 0.39 |
| **rep_96** | 0.87 | 0.92 | 0.90 | 0.91 | 0.25 | NA | 0.00 | 0.10 | 0.47 | 0.20 |
| **rep_97** | 0.99 | 0.98 | 0.94 | 0.94 | 0.94 | 0.72 | 0.31 | 0.32 | 0.13 | 0.23 |
| **rep_98** | 0.92 | 0.99 | 0.99 | 0.92 | 0.83 | 0.22 | 0.42 | 0.43 | 0.28 | NA |
| **rep_99** | 0.86 | 0.98 | 0.95 | 0.92 | 0.86 | 0.75 | 0.53 | 0.24 | 0.35 | 0.19 |
