# Supplementary material for: On taming the effect of transcript level intra-condition count variation during differential expression analysis: A story of dogs, foxes and wolves
Source: PLoS One. 2022 Sep 22;17(9):e0274591. doi: 10.1371/journal.pone.0274591 (PMC9498955; doi:10.1371/journal.pone.0274591)
Supplement: S6 Table — Number of transcripts kept and removed from the reference in each case study, wolves and dogs, and aggressive and tame foxes, across the filtered levels used (from the 99th to the 70th percentile). The first ten percentiles were explored in greater detail in steps of one, while the remaining were performed in steps of 5. (DOCX) [file pone.0274591.s013.docx]

|  | **Wolves and dogs** | | **Aggressive and tame foxes** | |
| --- | --- | --- | --- | --- |
| Percentile | Transcripts kept | Transcripts removed | Transcripts kept | Transcripts removed |
| 99 | 25923 | 184 | 25872 | 235 |
| 98 | 25601 | 506 | 25428 | 679 |
| 97 | 25253 | 854 | 25018 | 1089 |
| 96 | 24916 | 1191 | 24585 | 1522 |
| 95 | 24574 | 1533 | 24167 | 1940 |
| 94 | 24237 | 1870 | 23767 | 2340 |
| 93 | 23921 | 2186 | 23372 | 2735 |
| 92 | 23611 | 2496 | 22978 | 3129 |
| 91 | 23293 | 2814 | 22578 | 3529 |
| 90 | 22973 | 3134 | 22219 | 3888 |
| 85 | 21399 | 4708 | 20407 | 5700 |
| 80 | 19867 | 6240 | 18723 | 7384 |
| 75 | 18361 | 7746 | 17128 | 8979 |
| 70 | 16958 | 9149 | 15661 | 10446 |
