# Supplementary material for: On taming the effect of transcript level intra-condition count variation during differential expression analysis: A story of dogs, foxes and wolves
Source: PLoS One. 2022 Sep 22;17(9):e0274591. doi: 10.1371/journal.pone.0274591 (PMC9498955; doi:10.1371/journal.pone.0274591)
Supplement: S9 Table — Correlation values (r2) and the root mean square error (RMSE) from the regression analysis between the final dispersion estimates and the mean of normalized counts for both case studies, wolves and dogs, and aggressive and tame foxes. The number of outliers identified by DESeq2 are also presented. Values are shown for the non-filtered (NF) and all the filtered datasets used in differential expression analysis. (DOCX) [file pone.0274591.s016.docx]

|  | **Wolves and dogs** | | | **Aggressive and tame foxes** | | | |
| --- | --- | --- | --- | --- | --- | --- | --- |
| Percentile | Outliers | r^2^ | RMSE | Outliers | r^2^ |  | RMSE |
| NF | 281 | 0.74 | 0.81 | 20 | 0.49 |  | 1.12 |
| 99 | 262 | 0.76 | 0.79 | 20 | 0.5 |  | 1.11 |
| 98 | 249 | 0.77 | 0.77 | 19 | 0.51 |  | 1.11 |
| 97 | 233 | 0.78 | 0.76 | 18 | 0.52 |  | 1.1 |
| 96 | 219 | 0.78 | 0.76 | 18 | 0.53 |  | 1.1 |
| 95 | 218 | 0.79 | 0.75 | 17 | 0.54 |  | 1.09 |
| 94 | 205 | 0.8 | 0.74 | 16 | 0.55 |  | 1.09 |
| 93 | 198 | 0.8 | 0.73 | 15 | 0.55 |  | 1.09 |
| 92 | 186 | 0.81 | 0.73 | 15 | 0.56 |  | 1.08 |
| 91 | 179 | 0.81 | 0.72 | 14 | 0.57 |  | 1.08 |
| 90 | 172 | 0.82 | 0.72 | 14 | 0.58 |  | 1.07 |
| 85 | 141 | 0.83 | 0.7 | 13 | 0.61 |  | 1.05 |
| 80 | 123 | 0.85 | 0.68 | 13 | 0.65 |  | 1.03 |
| 75 | 118 | 0.86 | 0.67 | 14 | 0.68 |  | 1 |
| 70 | 102 | 0.87 | 0.65 | 15 | 0.72 |  | 0.97 |
