# Supplementary material for: On taming the effect of transcript level intra-condition count variation during differential expression analysis: A story of dogs, foxes and wolves
Source: PLoS One. 2022 Sep 22;17(9):e0274591. doi: 10.1371/journal.pone.0274591 (PMC9498955; doi:10.1371/journal.pone.0274591)
Supplement: S10 Table — List of the gene families, and shared genes, that were commonly regulated in dogs and tame foxes, using the non-filtered datasets. The number, and name, of the genes within each gene family are provided, with the corresponding log2fold-change values in brackets for each species. Within each family, single genes were charecterized as shared between dogs and tame foxes, or as exclusive to each of the two groups. When more than one transcript for a specific gene was present, all the log2FC values are reported. (DOCX) [file pone.0274591.s017.docx]

| **Gene Family** | **Group** | **N genes** | **Gene name and log2FC value** |  |
| --- | --- | --- | --- | --- |
| **Up regulated genes** | | | |  |
| Cholinergic receptor nicotinic alpha | **Shared** | 1 | **CHRNA5** (1.11 in dogs, 0.39 in tame foxes) |  |
| Squalene epoxidase | **Shared** | 1 | **SQLE** (0.55 in dogs, 0.29 in tame foxes) |  |
| Rho GTPase activating protein | **Shared** | 1 | **ARHGAP25** (0.86 in dogs, 0.70 in tame foxes) |  |
|  | Tame fox | 1 | ARHGAP30 (0.54) |  |
| Integrin alpha subunits | Dog | 3 | ITGA6 (1.26. 1.25); ITGA8 (1.14. 0.91); ITGAX (0.97) |  |
|  | Tame fox | 1 | ITGAL (0.71) |  |
|  | **Shared** | 1 | **ITGA7** (0.76 in dogs, 0.47 and 0.43 in tame foxes) |  |
| Myosin | Dog | 1 | MYO3A (1.12) |  |
|  | Tame fox | 2 | MYO1F (0.90); MYO1C (0.45) |  |
|  | **Shared** | 1 | **MYO7A** (0.82 in dogs, 0.39 in tame foxes) |  |
| Tribbles pseudokinase | Tame fox | 2 | TRIB1 (0.91); TRIB3 (0.75) |  |
|  | **Shared** | 1 | **TRIB2** (0.62 in dogs, 0.18 in tame foxes) |  |
| EF hand calcium binding | Dog | 1 | EFCAB1 (2.6) |  |
|  | Tame fox | 1 | EFCAB2 (0.43) |  |
| Transcription factor | Dog | 1 | TCF23 (2.05) |  |
|  | Tame fox | 1 | TCF19 (0.61) |  |
| Adhesion G protein-coupled receptors | Dog | 1 | ADGRG6 (1.46) |  |
|  | Tame fox | 1 | ADGRG1 (0.54) |  |
| Patatin Like Phospholipase Domain | Dog | 1 | PNPLA4 (1.42) |  |
|  | Tame fox | 1 | PNPLA7 (0.56) |  |
| SRY-box | Dog | 1 | SOX6 (1.27) |  |
|  | Tame fox | 1 | SOX17(0.81) |  |
| Hyaluronan and proteoglycan link protein | Dog | 1 | HAPLN1 (1.16) |  |
|  | Tame fox | 1 | HAPLN3 (0.67) |  |
| Serine/threonine kinase | Dog | 2 | STK17A (1.16. 1.14); STK32A (1.11) |  |
|  | Tame fox | 1 | STK40 (0.55) |  |
| Potassium channels | Dog | 1 | KCTD16 (0.98) |  |
|  | Tame fox | 1 | KCTD15 (0.69) |  |
| Podocalyxin like | Dog | 1 | PODXL (0.96. 0.85) |  |
|  | Tame fox | 1 | PODXL2 (0.77. 0.66. 0.64) |  |
| ATP binding cassette subfamily B | Dog | 1 | ABCB1 (0.93) |  |
|  | Tame fox | 1 | ABCB9 (0.50) |  |
| Zinc finger DHHC-type | Dog | 1 | ZDHHC15 (0.76) |  |
|  | Tame fox | 1 | ZDHHC1 (0.67) |  |
| Sushi domain | Dog | 1 | SUSD1 (0.69) |  |
|  | Tame fox | 1 | SUSD6 (0.44) |  |
| TBC1 domain family | Dog | 1 | TBC1D5 (0.55) |  |
|  | Tame fox | 1 | TBC1D7 (0.25) |  |
| Mitogen-activated protein kinase kinase kinases | Dog | 1 | MAP3K5 (0.51) |  |
|  | Tame fox | 1 | MAP3K11 (0.72) |  |
| Spermatogenesis Associated | Dog | 1 | SPATA5 (0.88) |  |
|  | Tame fox | 1 | SPATA24 (0.51) |  |
| **Down regulated genes** | | | |  |
| Stathmin domain | **Shared** | 1 | **STMND1** (-1.18 in dogs, -0.54 in tame foxes) |  |
| Oligoadenylate synthetase like | **Shared** | 1 | **OASL** (-0.40 in dogs, -0.54 in tame foxes) |  |
| Heat shock protein family B | Dog | 1 | HSPB8 (-0.69) |  |
|  | Tame fox | 1 | HSPB11 (-0.34) |  |
