# Supplementary material for: On taming the effect of transcript level intra-condition count variation during differential expression analysis: A story of dogs, foxes and wolves
Source: PLoS One. 2022 Sep 22;17(9):e0274591. doi: 10.1371/journal.pone.0274591 (PMC9498955; doi:10.1371/journal.pone.0274591)
Supplement: Attachment — Submitted filename: review_PONE-D-21-32424.docx [file pone.0274591.s018.docx]

Lobo et al present an evaluation of their software TVscript, which evaluates intra-condition variability in the counts that have been mapped to a transcriptome in an RNA-Seq experiment and removes the transcripts associated with the highest level of this variability, up to a user specified percentile threshold. They test the software by applying it to two pairs of datasets from wild and tame animals, wolves vs dogs and aggressive vs tame foxes. The greatest fraction of differentially expressed transcripts (DETs) is obtained by removing 3 to 5% of transcripts, and the authors describe some interesting features of the gene families of the corresponding differentially expressed genes, including common changes upon taming.

The approach to RNA-Seq analysis is a potentially interesting one, representing another approach similar in some ways to the “orthogonal filtering” of low-expressed transcripts that is commonly used to increase the power in the analysis of RNA-Seq experiments.

Unfortunately there are a number of aspects to the methodology that make it hard to recommend publication in the present form.

1 Most importantly, I don’t think the data sets analysed are appropriate for the main intention of the paper. It is hard to tell whether the alterations made to the transcriptome improve the results rather than inducing false positives. The data sets used for testing come from multiple batches, and two organisms, one of which is appreciably divergent from the transcriptome to which it is aligned. In short, there are too many other uncontrolled factors in the analysis done to tell whether the results are reliable. In testing TVscript, it would be better to use an approach like that taken in Rapaport et al 2013 (https://doi.org/10.1186/gb-2013-14-9-r95), which uses data sets where batch effects are better controlled, including one (GEO GSE 49712) where external rna control consortium (ERCC) spike ins were used to produce known true positive DEGs.

2 More detail is necessary about how the differential expression was performed; figure 2c seems to show that the dogs do separate by batch (1-5; 6; 7; 8-9) and one would normally use a design formula that took account of the different sources of the data, something like ~ batch + tameness.

(although the brain tissue and instrument used are also similar for dogs 6-9, so one could also try ~ tissue + tameness). The authors should state whether they used a formula like this, and justify why not if they did not (Ideally, the R script used for differential expression analysis could be made available).

I would also remark that, since the authors emphasise that data comes from different sources, it was not immediately clear, until I looked at supplementary table 1, that the wolves and dogs 1-5 all come from one study, and similarly all the foxes, aggressive and tame, come from one study. This should be brought out more in the text, as otherwise the reader is made to wonder how any difference between wild and tame will be detectable that is not confounded with batch effects.

3 It is unclear to me why the authors used the C. familiaris transcriptome for their work on the fox as well as the dog/wolf, when the fox and wolf lineages diverged 10 myr ago. A genome and transcriptome are available for the fox (https://www.ensembl.org/Vulpes_vulpes/Info/Index?db=core), and even though it is of lower quality than the dog, a higher mapping rate might have been expected. I appreciate that it makes the assessment of TVscript, and to some extent the comparison of dog and fox DET results, more straightforward (though information on orthology is also available). The low mapping rate and slightly strange clustering of the points in the PCA plot fig 2d are indications there may be some problems with the fox data that might in part come from the choice of transcriptome, and this casts some doubt on the DET results for me.

My recommendation would be to split the work into two papers, one comparing the wild and tame animals, which to me was the most interesting part of the manuscript, and one assessing TVscript. It appears to me from comparing Tables 1 and 2 in the paper (filtered transcriptome) with supplementary table 6 (unfiltered transcriptome), that the filtering did not make a very big difference here. Hence the first paper could use the results from the more standard methods of supp. Table 6 and the interesting overlaps between the changes on domestication in the two pairs of animals would still be largely maintained. The second paper really would really need to use different test data sets , as suggested above, to establish whether TVscript is genuinely increasing sensitivity without introducing type I errors.

I would like to thank the authors for preparing the manuscript carefully, providing detailed results and supplementary material, and providing access to the code of TVScript along with links to other useful material on sourceforge.
