# Supplementary material for: On taming the effect of transcript level intra-condition count variation during differential expression analysis: A story of dogs, foxes and wolves
Source: PLoS One. 2022 Sep 22;17(9):e0274591. doi: 10.1371/journal.pone.0274591 (PMC9498955; doi:10.1371/journal.pone.0274591)
Supplement: Attachment — Submitted filename: response_to_reviewers.docx [file pone.0274591.s020.docx]

**Reviewer #1**

“I commend the authors for the comprehensive amendments and explanations. I think the article is in great shape. The provision of scripts and data on zenodo is appreciated. Please consider the following points as optional suggestions.”

We thank the reviewer for these minor revisions and we have adjusted the manuscript accordingly. The time and effort that the reviewer has spent on this, and on the previous, round of review has been invaluable for our manuscript.

“1. This new simulation is a welcome addition that supports the perceived need for a tool like TVscript.In figure 2, I would recommend putting the legend for light and dark grey boxes on the plot itself.”

We have now added a key to figure 2 explaining the light and dark grey boxes. This has also been added to the similar figure S5.

“2. OK
3. OK
4. OK

5. OK, but the passage on line 723 should be written in a clearer, more straightforward way.”

We have reworded the three lines following line 723.

“6. OK.

7. OK, but "high r2 correlation values" should be qualified with a specific range (eg: 85-98%), so that the reader can understand what "high" means.”

We have now added the range to the text (on line 405) by saying: “R^2^ values ranged between 0.8546 and 0.9944.”

“8. OK
9. OK.
10. OK

Typo: "we also had an interested in understanding whether"”

Corrected

**Reviewer #2**

“I am grateful to Lobo et al for their efforts in addressing my criticisms of the first version of their manuscript.”

We are very grateful for the comments provided by this reviewer that, in conjunction to those provided by the first reviewer, has greatly clarified and improved our work.

“1 Appropriateness of data sets used to assessing TVscript: I think that by using extensive simulated data the assessment of the behaviour of TVscript is much improved.”

Agreed. The simulations allowed us to test our approach within a highly controlled framework prior to application on the real data.

“2 more detail has been given on how the DE analysis was performed as requested. I am not totally convinced by the lengthy discussion of unknown effects (though there is no reason to remove it); clearly there are always unknown factors, but that does not affect the apparent effect of batch in the PCA plots. But in any case, the important test, that including batch in the statistical model for DE in the dogs, has been done and the results (fig S6) seem to confirm that it does not have a very large effect”

Testing for batch effects was an important addition that we had not initially included. We feel that the inclusion of this, along with the results based on simulations (where batch effects less relevant), has greatly improved completion when presenting our results.

“3. I thank the authors for checking the mapping rate of the fox data to the fox transcriptome (and, incidentally, by mapping with kallisto as well). Even though it turned out not to affect the mapping rate very much, I feel this was an important check to perform.”

This was an important check that we should have provided within the first version in order to set the mind of readers at rest relative to the question raised by the reviewer. We feel that it is an interesting point and we are pleased to have made this addition at the reviewer’s suggestion.

“4. I appreciate that factors outside the authors’ control can affect the way that a project ends up being carried out and written up. Although my suggestion was to divide the manuscript, I do not insist on it, I am content with the manuscript’s current form. The criticisms that I made in my first review, at least, are allayed.”

Great, we completely understood why the reviewer was thinking about two separated papers here, and we will still aim to produce a future paper with our own generated RNA-seq datasets.

“However the first reviewer raised other serious points particularly point 5 about filtering where the sample group information is used (see Bourgon, Gentleman, and Huber 2010). I did not spot this in my own review and it is for the first reviewer to assess whether the additional simulations have addressed this satisfactorily. I did notice that in the discussion of the matter in the DESeq2 vignette (http://bioconductor.org/packages/devel/bioc/vignettes/DESeq2/inst/doc/DESeq2.html#independent-filtering-and-multiple-testing) a histogram of the p-value of the filtered genes is provided, showing that it is approximately uniform. I would tentatively suggest (again, I defer to the first reviewer here) that, done for transcripts rather than genes, a p-value histogram could provide an empirical way of demonstrating that the filtering is independent of the test statistic under the null hypothesis, if required.”

Reviewer 1 was happy with our response to their point 5 and did not require any further clarification. Given that reviewer 2 has stated that “… I defer to the first reviewer here …” we feel that point 5 has been sufficiently discussed within the manuscript for the scope of our study.
